# COVID-19: Reduced Lung Function and Increased Psycho-emotional Stress

**DOI:** 10.6026/97320630016293

**Published:** 2020-04-30

**Authors:** Dongyuan Wu, Dorothy Ellis, Susmita Datta

**Affiliations:** 1 Department of Biostatistics, College of Public Health and Health Professions College of Medicine University of Florida 2004 Mowry Rd, 5th Floor CTRB, P.O. Box 117450 Gainesville, FL 32611-7450

**Keywords:** COVID-19, Lung Function, Psycho-emotional Stress

## Abstract

The COVID-19 outbreak causing reduced lung function and increased psycho-emotional stress in the community worldwide. Therefore, it is of interest to document such viral outbreak related emotional stress data in the community with known molecular and patho-physiological parameters of the affected individuals. We provide a concise, coherent, critical, precise, specific and direct narration of such events from a community research viewpoint using known molecular data in this editorial.

## Description:

COVID-19 is a global pandemic-affecting individual in 202 countries as of March 28, 2020 [1]. Characteristics of COVID-19 include fever, non-productive cough, dyspnea, myalgia, fatigue, normal or decreased leukocyte counts, and radiographic evidence of pneumonia [16]. In severe cases, shock, acute respiratory distress syndrome (ARDS), acute cardiac injury, acute kidney injury, and death can occur [16]. There is also evidence of potential for long-term lung dysfunction in COVID-19 survivors; a prospective longitudinal study of 90 patients with pneumonia due to COVID-19 found that 94% of discharged patients still had evidence of disease on their final CT scans [17]. This included the persistence of ground-glass opacity (GGO), which, in some patient, increased as the patient recovered enough to be discharged [17]. GGO are hazy white opaque structures found on CT scans, which do not obscure underlying bronchial structures or pulmonary vessels [7]. GGO also increased as SARS patients recovered [17]. While it is now too early to determine whether current COVID-19 patients will experience long-term lung dysfunction, some SARS patients experienced permanent decreased lung function [6].

It follows then that in addition to the anxiety many experience from worrying about themselves, family members, or friends contracting COVID-19 or worrying about economic fallout and social isolation from COVID-19 social distancing measures, some survivors of COVID-19 may also have to cope with long-term lung dysfunction. There is evidence of an association between reduced lung function and stress [9] or mental health problems [2]. A study using data from the first National Health and Nutrition Examination Survey found that restrictive and obstructive lung function were associated with significantly increased likelihood of mental health problems and significantly lower overall well-being [2]. We wanted to explore whether there is evidence of an association between reduced lung function and stress or mental illness at a cellular level.To investigate the relationship between decreased lung function and stress levels, we used a single cell RNA sequencing (sc-RNA-seq) dataset, which consisted of lung tissue samples, collected from the lungs of 9 individuals with pulmonary fibrosis and 8 lung donors. Pulmonary fibrosis is a disease in which fibrotic scarring replaces the alveolar tissue of the lung and leads to difficulty breathing and eventual respiratory failure [13]. We performed deferential expression analysis using the Model-based Analysis of Single Cell Transcriptomics (MAST) package in R [11]. The initial study using these data explored creating a cell atlas for pulmonary fibrosis [13]. In a later study, the data from the lung donors were used to profile Angiotensin-converting enzyme 2 (ACE2). ACE2 is the receptor for SARS-COV, the virus that causes SARS, and is also believed to be the receptor for SARS-COV-2, the virus that causes COVID-19 [18]. While this paper used donor lungs to investigate the function of ACE2, we wanted to determine whether there is evidence of deferential expression of genes between the idiopathic pulmonary fibrosis (IPF) and healthy donors and whether these genes are related to systemic stress responses or psychological disorders. Due to time limitations, we performed our analysis on a random subset; we used five individuals from the pulmonary fibrosis group and four individuals from the donor group. For the pulmonary fibrosis group, we used only individuals who suffer from IPF. To inspect the data visually, we took some exploratory steps, including the figure below. This figure represents side-by-side plots of the clusters of cells using the Rtsne implementation of t-distributed stochastic neighbour embedding (t-SNE) [8]. From the plot, we can see that there is a significant difference in the grouping of cells between the healthy donor and the IPF groups.

After using MAST to identify deferentially expressed genes, we used the Database for Annotation, Visualization and Integrated Discovery (DAVID) [4- 5] to annotate the functionality of these genes. We identified 24 functional annotation clusters that were significant (FDR <0.05). Of these, we identified 11 top functional clusters that had evidence of an association with stress or mental illness. These top 11 annotations were related to cell-cell adherens junctions (GO:0005913), cadherin binding involved in cell-cell adhesion (GO:0098641), cell-cell adhesion (GO:0098609), peptide antigen binding (GO:0042605), antigen processing and presentation (GO:0019882), antigen processing and presentation of peptide antigen via MHC (GO:0002474 [class I], GO:0002479 [class I TAP-dependent], GO:0019886[class II]), MHC class II protein complex binding (GO:0023026), MHC class II protein complex (GO:0042613), and response to reactive oxygen species (GO:0000302).

First, cellular adhesion has been associated with acute psychological stress [12]. Cadherin genes, which are genes involved in the adherens junction, cadherin binding, and cell-cell adhesion processes and bind cells within tissues, are associated with major psychiatric disorders [3]. Of the processes identified here, GO:0098609 specifically has been implicated as having a significant association with psychiatric illness [3]. Cell-cell junction pathways are also associated with depression and schizophrenia [3]. Stress has an effect on many biological functions; the functions related to antigen processing and MHC identified above are impaired by cortisol, a hormone released as a response to psychological stress [15]. Antigen processing and presentation play a significant role in the body's immune response by allowing immune cells to recognize pathogens. Without these processes, immune cells would unable to clear infected cells [15]. The function GO:0042605 specifically was also identified as a candidate pathway for schizophrenia [10]. Finally, reactive oxygen species (ROS) causes oxidative stress, which, in turn, is associated with stress response and with the development of mental disorders including depression and anxiety [14].

## Concluding Remarks:

While some of the gene functions identified by this analysis may not be deferentially expressed in individuals with COVID-19, many of the functions identified by this analysis appear to be generally associated with immune response and lung function. The disruptions in normal lung function identified here may also apply to the long-term effects of COVID-19 in some recovered individuals. There is a body of evidence for the existence of relationships between psychological stress and mental disorders and immune response, oxidative stress, and lung function. It is important not only to focus on the recovery from acute disease but also to focus on the psychological impact of the potential for long-term disability due to permanent loss of lung function in survivors of COVID-19.

## Figures and Tables

**Figure 1:**
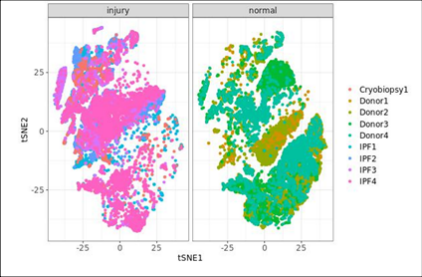
Side by side plot of first two t-SNE components
